# Context is key: glucocorticoid receptor and corticosteroid therapeutics in outcomes after traumatic brain injury

**DOI:** 10.3389/fncel.2024.1351685

**Published:** 2024-03-11

**Authors:** Morgan A. Taylor, Olga N. Kokiko-Cochran

**Affiliations:** Department of Neuroscience, Chronic Brain Injury Program, Institute for Behavioral Medicine Research, College of Medicine, The Ohio State University, Columbus, OH, United States

**Keywords:** traumatic brain injury, neuroinflammation, glucocorticoid, glucocorticoid receptor, stress, microglia

## Abstract

Traumatic brain injury (TBI) is a global health burden, and survivors suffer functional and psychiatric consequences that can persist long after injury. TBI induces a physiological stress response by activating the hypothalamic-pituitary-adrenal (HPA) axis, but the effects of injury on the stress response become more complex in the long term. Clinical and experimental evidence suggests long lasting dysfunction of the stress response after TBI. Additionally, pre- and post-injury stress both have negative impacts on outcome following TBI. This bidirectional relationship between stress and injury impedes recovery and exacerbates TBI-induced psychiatric and cognitive dysfunction. Previous clinical and experimental studies have explored the use of synthetic glucocorticoids as a therapeutic for stress-related TBI outcomes, but these have yielded mixed results. Furthermore, long-term steroid treatment is associated with multiple negative side effects. There is a pressing need for alternative approaches that improve stress functionality after TBI. Glucocorticoid receptor (GR) has been identified as a fundamental link between stress and immune responses, and preclinical evidence suggests GR plays an important role in microglia-mediated outcomes after TBI and other neuroinflammatory conditions. In this review, we will summarize GR-mediated stress dysfunction after TBI, highlighting the role of microglia. We will discuss recent studies which target microglial GR in the context of stress and injury, and we suggest that cell-specific GR interventions may be a promising strategy for long-term TBI pathophysiology.

## 1 Introduction

Traumatic brain injury (TBI) is a major source of injury-related disability and death across the globe (Maas et al., 2017). A study on the Global Burden of Disease estimates that over 20 million individuals suffer from TBI each year (Mathers, 2016). The severity of injury varies depending upon the source of the trauma, but broadly a TBI is caused by force to the head that disrupts normal brain function (Faul and Coronado, 2015). Death rates due to TBI have declined with advances in medical facilities, but millions of survivors experience long-term effects of injury (Coronado et al., 2012). The initial, or primary, injury includes cell damage and hemorrhage resulting from the mechanical forces of TBI (Ng and Lee, 2019). The degree of primary injury depends on the severity and type of TBI. This transitions to prolonged secondary damage that can persist for years.

As a result of injury-induced damage, TBI survivors may suffer a number of debilitating physical, cognitive, and psychiatric consequences. Neuroinflammation is a major culprit of secondary damage after TBI, driven by infiltration of peripheral immune cells and enhanced reactivity of brain resident microglia (Ramlackhansingh et al., 2011; van Vliet et al., 2020; Witcher et al., 2021). Prolonged neuroinflammatory damage after TBI perpetuates chronic dysfunction of the stress response. For example, TBI induces significant alterations in basal levels of major stress hormones, and clinical evidence suggests a loss of circadian cortisol rhythms in TBI survivors (Llompart-Pou et al., 2010; Griesbach et al., 2011). In addition to this baseline dysfunction, multiple studies indicate significant alterations in the hormonal response to external stressors following TBI. Heightened reactivity to acute stress has been reported in the 1st weeks after lateral fluid percussion injury (FPI) in rats, indicated by exaggerated stress-induced hormone production (Griesbach et al., 2011). In contrast, a blunted response to stress has been reported at longer (3–6 weeks) time points after TBI (Taylor et al., 2006, 2013). This dysfunction in both baseline hormone levels and stress reactivity is clinically significant, as TBI survivors are highly susceptible to secondary stressors such as insomnia, depression, chronic pain, and medical-related anxiety (Jain et al., 2014; Agtarap et al., 2021). Impaired stress signaling can make it increasingly challenging to maintain homeostasis after stress, exacerbating stress- and injury-induced outcomes and negatively impacting quality of life (Gilis-Januszewska et al., 2020).

Previous studies suggest that anti-inflammatory treatments could help improve recovery after TBI, further emphasizing the underlying central role of neuroinflammation in TBI pathophysiology (Xu et al., 2017; Chen et al., 2018; Wei et al., 2021). However, full recovery after injury remains elusive for many TBI survivors. Many are highly vulnerable to secondary forms of stress, including medical-related psychological stress, depression, and insomnia (Agtarap et al., 2021). These factors not only make adjusting to life after TBI difficult for patients, but also challenge the body's stress response and synthesize with pathophysiology of the injury. This exacerbates chronic inflammation and impedes recovery.

Glucocorticoids (GC) are endogenous steroid hormones produced in response to stress. GCs bind to glucocorticoid receptors (GR) expressed throughout the body to regulate the immune and inflammatory response through a multitude of downstream effectors. Natural and synthetic GCs have been used as a means of treating side effects of inflammation, such as edema and intracranial pressure (ICP) acutely after TBI. However, mixed levels of success and no overall effect on patient survival has diminished enthusiasm for therapeutic relevance with this approach (Olldashi et al., 2004; Edwards et al., 2005). Furthermore, a major drawback to long-term GC use is a high risk of systemic side effects, including osteoporosis and immunosuppression. Modern developments in biomedical technologies are being applied in preclinical models to administer GCs without the risk of systemic effects, but these are preliminary studies with limited potential for clinical application as of yet.

More recently, other methods of targeted GR manipulation have been explored to affect chronic neuroinflammatory and behavioral outcomes in rodent models of TBI. Many of these studies make use of RU486 (mifepristone), a potent GR antagonist, but RU486 effects on progesterone receptors limit its translational appeal (Meyer et al., 1990). Others have employed novel genetic techniques to deplete GR in specific cell and tissue types. A few of these studies manipulate GR in the context of TBI, and some inconsistencies between studies suggest that the timing of manipulation, injury model, and cell type play a major role in outcome. Together, these studies suggest that GR impacts stress-related neuroinflammation driven by microglia and give promise to a potential avenue for repurposing GC intervention after TBI.

In this review, we will summarize GR-mediated outcomes after TBI. First, we will discuss the damage sustained by TBI, and how this stimulates the physiological stress response. Second we will describe the long-term effects of TBI on the stress response and glucocorticoid signaling, as well as the effects of additional stress on recovery from TBI. We will review early GC-based TBI therapeutics and discuss some of the limitations of these previous studies. Then, we will establish that the roles of GC and GR are complex and highly context-specific, and we suggest that a localized cell-specific therapeutic approach may be a more promising focus for future studies. Finally, we will review recent studies involving GR manipulation and emphasize the effects on microglia activation and microglia-associated neuroinflammation. Given the detrimental outcomes associated with previous clinical studies, synthetic GCs are no longer recommended as a post-TBI treatment. However, neuroendocrine dysfunction after TBI remains a significant health burden that could be exaggerated by secondary stressors. We will summarize what is known about GC and GR after TBI and discuss recent strategies for studying their context-specific roles. In conclusion, cell-specific GC intervention remains an underexplored but promising direction for future study.

## 2 Mechanisms of damage after TBI

**Figure 1:**
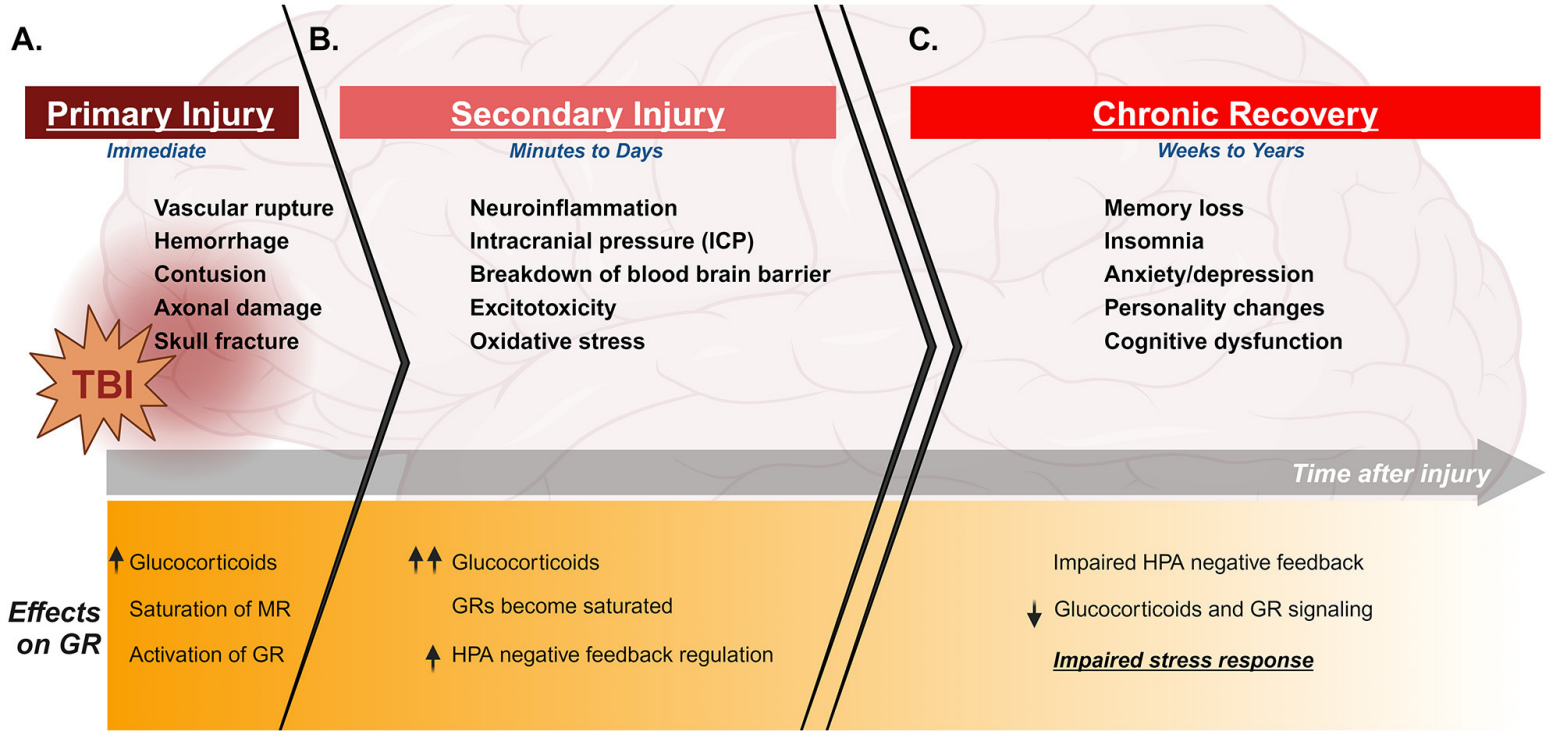
Timeline of clinical features and stress effects of TBI. Top panels indicate clinical features of TBI during primary injury, secondary injury, and chronic recovery. Lower panels describe effects of injury on the stress response and GR during the same phases of injury. TBI immediately induces tissue and vascular damage and stimulates the HPA axis to cause glucocorticoid release and activation of GR **(A)**. The damage in the brain progresses to secondary injury with increased neuroinflammation and building intracranial pressure, as well as varying degrees of damage to blood brain barrier and excitotoxicity depending on the injury. This secondary damage can persist and progress for several days, during which glucocorticoid levels continue to build and GR becomes saturated **(B)**. This leads to an increase in negative feedback regulation of the HPA axis. TBI survivors can experience chronic consequences for several weeks to years after injury, including cognitive and psychiatric dysfunction. Importantly, TBI also induces chronic neuroendocrine dysfunction over this period of time, which can include an aberrant increase in negative feedback suppression of the HPA axis. This results in an impaired stress response, with reduced glucocorticoid production and GR signaling even in the presence of additional stress **(C)**. Created with BioRender.com.

TBI-induced damage to the brain is categorized into immediate and delayed mechanisms. The primary injury is the immediate damage directly resulting from physical impact to the head. The scale of primary damage varies depending on the type and severity of TBI sustained. This can include varying degrees of localized axonal damage, hemorrhage, and edema (Figure 1A; Zemlan et al., 1999; Ng and Lee, 2019; Sabet et al., 2021). The effects of primary injury transition over a period of hours or days to more prolonged damage. This is referred to as the secondary injury, which can persist for years. Long-term secondary injury is perpetuated by several injury-induced mechanisms, including neuroinflammation and excitotoxicity (Figure 1B; Ng and Lee, 2019). Importantly, injury-induced damage often leads to chronic cognitive and psychiatric consequences which can persist for years (Figure 1C).

### 2.1 TBI stimulates the HPA axis

Any perceived psychological or physical stress, including an injury such as TBI, initiates a physiological response in the body to maintain homeostasis. The hypothalamic-pituitary-adrenal (HPA) axis is a major element of the stress response. Stressful stimulus causes activation of the hypothalamic paraventricular nucleus (PVN). Cells in the PVN secrete corticotropin releasing hormone (CRH), which stimulates the anterior pituitary to produce adrenocorticotropin hormone (ACTH). ACTH is secreted and signals to the adrenal glands, resulting in corticosteroid synthesis (Tapp et al., 2019; Leistner and Menke, 2020). Corticosteroids are steroid hormones that act throughout the body to regulate homeostasis in response to stress, contributing to fundamental biological processes in all major tissue types (HPA axis has been recently reviewed in detail by Leistner and Menke, 2020).

GCs are a major class of corticosteroids released through HPA axis signaling. They are essential stress-response hormones, playing key roles in a variety of physiological processes including metabolism, immunity, and cognition (Fietta et al., 2009; Cain and Cidlowski, 2017). Cortisol is the main glucocorticoid in humans, while corticosterone is the glucocorticoid in most other animals, including rodents (Katsu and Baker, 2021). Primary injury stimulates immediate activation of the stress response through stimulation of the HPA axis, and clinical studies show elevated serum cortisol in the first few days following TBI (Figure 1A; Woolf, 1992; Wagner et al., 2011).

### 2.2 TBI induces chronic neuroinflammatory damage

The severe tissue damage sustained at the site of primary injury induces a rapid inflammatory response, with significantly increased cytokine production and microglia activation detected in experimental models 1 day after TBI (Tobin et al., 2014; Witcher et al., 2021). Cellular and vascular damage often leads to breakdown of the blood brain barrier (BBB) in the first several days following TBI (reviewed by Cash and Theus, 2020). This BBB disruption allows peripheral immune cells to enter the injured brain, synthesizing with the already inflamed resident cells in the brain to result in additional cytokine production and heightened inflammatory state (Sabet et al., 2021). Neuroinflammation underlies much of the secondary damage sustained after TBI. In addition to long-term outcomes such as cognitive and behavioral consequences, chronic neuroinflammation can also result in severe clinical symptoms such as intracranial pressure (ICP) and edema (Figure 1B). Importantly, cerebral edema not only causes severe pain and discomfort for patients but is also one of the major factors in survival after TBI (reviewed by Zusman et al., 2020).

## 3 Stress and TBI activate glucocorticoid receptor signaling

**Figure 2:**
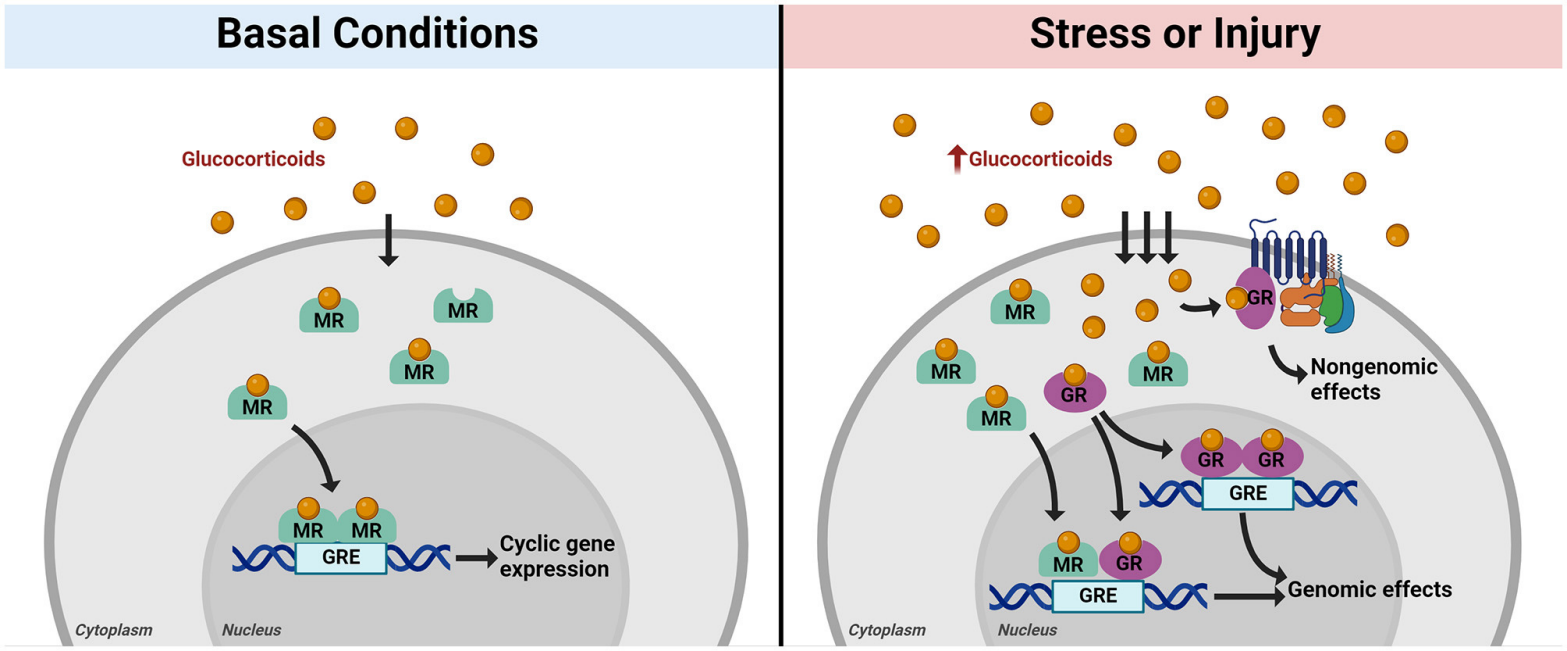
Mechanisms of intracellular glucocorticoid receptor action. Glucocorticoids (GC) diffuse through the cell membrane and enter the cytoplasm, where they are taken up by intracellular mineralocorticoid receptors (MR, in green) or glucocorticoid receptors (GR, in purple). Under basal conditions, MR binds to GC as levels fluctuate throughout the day in ultradian rhythm. Ligand-bound MR then translocates into the nucleus to effect cyclic gene expression and maintain homeostasis through interactions between dimerized MR and glucocorticoid response elements (GREs) in DNA **(left panel)**. When the stress response is activated, GC production increases and more GC diffuses into the cell. MRs become saturated, and GRs begin to bind GCs. GR exerts rapid effects in response to increased GC through interactions with membrane-bound receptors (non-genomic effects). Ligand-bound GR also translocates into the nucleus along with MR, where it forms heterodimers with MR or forms GR homodimers to effect gene expression in response to stress [genomic effects, **(right panel)**]. Created with BioRender.com.

GCs bind intracellular mineralocorticoid (MR) and glucocorticoid receptors (GR) to affect numerous signaling pathways. Both receptor types are expressed in almost all tissues throughout the body, including the brain. MRs are high-affinity receptors that bind GCs under basal conditions, while low-affinity GRs are recruited as GC levels increase under conditions of stress. Both receptors act as ligand-dependent transcription factors, translocating into the nucleus upon ligand binding to affect gene expression. GCs are released in an ultradian rhythm, with distinct peaks occurring at regular intervals over each 24-h period (Lightman et al., 2008). A primary function of MR is to regulate HPA axis activity throughout the day by binding GC in response to ultradian pulses, translocating into the nucleus to regulate cyclic gene expression (Figure 2, left panel; Herman et al., 2016). In contrast, GR has a lower binding affinity for GC and for the most part remains in the cytoplasm under basal conditions (Conway-Campbell et al., 2007). Once a threshold concentration of GC is reached, GR is activated. In general, this occurs following a stressor or injury which induces HPA axis activation (Figure 2, right panel). Clinical and experimental evidence indicates similar HPA activation and GC release in the immediate response to TBI (Figure 1A; Woolf, 1992; Lu et al., 2009; Wagner et al., 2011).

GR exerts a vast range of effects throughout the body, affecting multiple cell and tissue types and impacting diverse functional and behavioral responses. A major function of GR is to mediate negative feedback regulation of the HPA axis. As lower-affinity MRs become saturated with stress-induced increases in GC, GRs become activated and work to turn GC production back down (Figure 1B). GR expressed in cells of the PVN, anterior pituitary, and adrenal glands bind GC at these higher concentrations, resulting in inhibition of output at every level of the HPA axis (Kim and Iremonger, 2019; Shipston, 2022). This is a critical component of HPA axis functionality, reducing production of ACTH and CRH and preventing excessive corticosteroid release over extended periods of time in order to maintain homeostasis after exposure to stress. Importantly, feedback mechanisms are crucial for turning the stress response down after a perceived threat has passed. Impaired feedback can result in overactive stress response and negative impacts to health (reviewed by Kim and Iremonger, 2019).

### 3.1 Individual roles of distinct GR isoforms are understudied

The human glucocorticoid receptor gene, *Nr3c1*, is alternatively spliced into several variants. The two most well-studied isoforms are GRα and GRβ. GRα is the more widely expressed ligand-binding product, regulating several key homeostatic processes (Meduri and Chrousos, 2020). Expression of GRβ is more limited, but it is a component of several intracellular complexes, including a heterodimer with ligand-bound GRα (De Castro et al., 1996). GRβ also plays a key role in repressing some of the transcriptional effects of GRα (Ramos-Ramírez and Tliba, 2021). Interestingly, *in vitro* experiments suggest that GRβ plays a role in astrocyte-mediated wound healing, but the specific role of GRβ in the context of TBI has not been explored (Yin et al., 2013; Wang et al., 2015). Rodents also produce different isoforms of GR through alternative splicing of *Nr3c1*, though the precise mechanisms of splicing differ from those in humans. The more limited rodent GRβ isoform was not identified until 2010, but recent evidence suggests that, like its human homolog, it is important for negative regulation of many of the effects of GRα (Hinds et al., 2010; Dubois et al., 2013). In both humans and rodents GRα is the classic form of GR that responds to glucocorticoids (Nicolaides et al., 2010). However, because the discovery of GRβ in rodents is relatively recent, there is rarely a distinction between the α and β isoforms in preclinical literature. Therefore, for the purposes of this review, “GR” will collectively refer to either isoform produced by the *Nr3c1* gene. Still, it is important to note that this is an oversimplification. Though GRα is the more prevalent isoform, it is possible that GRβ plays a role in the response to TBI, perhaps through astrocyte-mediated mechanisms similar to those described in the context of wound healing (Yin et al., 2013; Wang et al., 2015). Future studies should be careful to make the distinction between GRα and GRβ and consider that they may have very different injury-related roles.

### 3.2 Genomic and non-genomic mechanisms of GR after TBI have not been characterized

There are two distinct categories of GR actions: fast (non-genomic) mechanisms and delayed (genomic) mechanisms (Figure 2, right panel). Non-genomic, rapid effects of GR work through interactions with membrane-associated G protein-coupled receptors (GPCRs; Tasker et al., 2006). Many of these rapid effects rely on receptor-dependent kinase activity (Oakley and Cidlowski, 2013). Non-genomic mechanisms have a broad range of functional outcomes throughout multiple tissues, from contraction of smooth muscle in the trachea to pancreatic insulin release (Se Sutter-Dub, 2002; Sun et al., 2006). In the brain, GR rapidly affects neuronal synapse plasticity and transmission, with detectable changes in neuronal activity occurring in just a few minutes of GR activation (summarized in Myers et al., 2014). Synthetic glucocorticoids such as dexamethasone are frequently used in experimental studies to activate GR and are instrumental in elucidating mechanisms of GR activity. Previous experimental studies show that a single injection of synthetic GC in rats causes a rapid increase (within 7.5–15 min) in the locomotor response to a novel environment (Sandi, 1996). Patch clamp studies in mice suggest dexamethasone induces rapid GR-mediated effects on both excitatory and inhibitory synapses (Nahar et al., 2015).

GR is primarily studied in the context of its delayed transcriptional effects, though these are complex and multifaceted. Upon ligand binding, cytoplasmic GR translocates into the nucleus, where it regulates transcription of hundreds of anti- and pro-inflammatory genes. GR can both activate and repress transcription of its downstream targets, either through direct binding of glucocorticoid response elements (GREs) in DNA or by tethering to other regulatory DNA-binding proteins. In the nucleus, GR can form a heterodimer with MR, or it can homodimerize and bind to other GREs to affect stress-related gene expression (Figure 1, right panel). GR may act as an anti- or pro-inflammatory regulator under different conditions, but it is notorious its powerful anti-inflammatory immunosuppressive effects in response to stress. Many of these anti-inflammatory transcriptional effects occur through tethering of GR to pro-inflammatory factors, such as Nuclear Factor κB (NFκB) or Activator Protein 1 (AP-1), inhibiting their transcription to promote a return to homeostasis (reviewed in Chinenov et al., 2013).

Importantly, of the limited experimental studies on the effects of TBI on *Nr3c1*/GR, the majority do not dissect the effects on specific downstream mechanisms of GR. Some studies quantify nuclear GR as a measure of transcriptional activity (Zhang et al., 2021). Others measure GC-dependent downstream targets, such as *Sgk1* or *FKBP5*, as markers of transcriptional activation of GR (Aminyavari et al., 2019; Lengel et al., 2022). Still, the use of these methods to measure genomic GR activity in the context of TBI have been very limited to date. Furthermore, there is a stark lack of studies exploring the rapid, surface receptor-mediated mechanisms of GR after TBI, and these may be worth a closer look. For example, previous work has linked GR to N-methyl-D-aspartate receptor (NMDAR) activity in the hippocampus, which is known to contribute to TBI-induced excitotoxicity and cognitive deficits (Zhang et al., 2012; Baracaldo-Santamaría et al., 2022). NMDAR agonists have shown promise in improving cognition after TBI, but extensive issues regarding their safety and efficacy have limited their therapeutic potential and stalled clinical studies (Khormali et al., 2022; Hanson et al., 2024). A closer look at the role of GR in activity of NMDAR and other surface receptors may shed light on previously unexplored mechanisms of excitotoxicity after TBI.

## 4 TBI induces neuroendocrine dysfunction

The prolonged neuroinflammatory damage that perpetuates during secondary injury provokes robust alterations in stress signaling, impairing HPA axis function as the injury progresses. One possible mechanism is altered negative feedback signaling, resulting in aberrant suppression of GC (Figure 1C). Preclinical models indicate changes in baseline stress hormone levels, such as elevated ACTH and reduced corticosterone within the 1st weeks after injury compared to controls (Griesbach et al., 2011). HPA axis dysfunction, including hypopituitarism and acute secondary adrenal insufficiency, are common in human TBI survivors (Bondanelli et al., 2004, 2005; Agha et al., 2007; Gilis-Januszewska et al., 2020). Additional clinical studies suggest that cortisol circadian rhythms are lost in patients after TBI (Llompart-Pou et al., 2010). Together, these studies point to TBI-induced alterations in baseline neuroendocrine function.

In addition to baseline dysfunction, multiple studies indicate significant alterations in the response to external stressors following TBI. Experimental evidence suggests altered stress signaling, including suppression of GR, in the sub-acute phase after injury (reviewed by Hoffman and Taylor, 2019). A study on the post-injury response to acute stress suggested a heightened stress response in the 1st week following mild fluid percussion injury (FPI), with increased reactivity of corticosterone and ACTH after post-injury acute stress compared to non-injured animals (Griesbach et al., 2011). However, a subsequent study using the controlled cortical impact (CCI) model of injury suggested that there was a blunted stress response at 7 days post injury (DPI; Taylor et al., 2008). Additional studies have reported differential post-TBI effects on the HPA axis in male and female rats (Russell et al., 2018; Bromberg et al., 2020). A blunted response to stress has been reported at longer (3–6 weeks) time points after TBI, and previous studies suggest that GR activity may play a role in this (Taylor et al., 2006, 2013).

### 4.1 Neuroendocrine dysfunction exacerbates consequences of TBI

Even after the initial recovery from TBI, many survivors experience lasting symptoms which can continue for several months or even years after injury. These include behavioral dysfunction such as depression or anxiety, and cognitive issues such as attention disorders and memory impairment (reviewed by Karr et al., 2014; Howlett et al., 2022). Given the robust effects of TBI on the physiological stress response it comes as no surprise that stress also profoundly impacts long-term recovery after TBI, but the relationship between injury and stress is complex. Persistent or intense stress is harmful and can exacerbate neurological dysfunction. However, the mechanisms through which stress affects post-injury recovery depend on the timing and type of stressor, and some studies suggest that stress can even improve outcomes after TBI (reviewed by Houle and Kokiko-Cochran, 2022; Zheng et al., 2022).

### 4.2 Pre-injury stress alters the response to TBI

TBI often occurs in high stress environments, including domestic violence, combat, and sporting events (Brand et al., 2023). Under these conditions, GC levels are already elevated prior to injury, and MRs become saturated while GRs are overactivated (Fox et al., 2016). Multiple previous studies have indicated that pre-injury stress can significantly impact outcomes after TBI. Early life stress (ELS) paradigms are often used in preclinical studies to model physically and psychologically stressful life events. Maternal separation, in which pups are temporarily separated from their mothers for an extended period, is a common method of ELS, and it has been shown to exacerbate TBI-induced cortical atrophy and learning deficits in a rodent FPI model (Sanchez et al., 2021). Other experimental stress paradigms, such as chronic unpredictable stress (CUS), have also been shown to exacerbate subsequent injury. Mice exposed to 5 weeks of mild CUS followed by CCI exhibit exaggerated learning and memory deficits compared to mice receiving CCI in the absence of additional stress (Park et al., 2023). One proposed mechanism for this stress-induced aggravation of TBI pathology is that existing stress primes the immune system, leading to an exaggerated inflammatory response to a subsequent TBI (Brand et al., 2023). Indeed, ELS is correlated with higher TBI-induced microglial activation and production of inflammatory cytokines (Lajud et al., 2021; Sanchez et al., 2021). These studies indicate that pre-injury stress exacerbates cognitive and inflammatory consequences of TBI, though the mechanisms remain unclear. A likely explanation is that pre-existing stress leads to a dysfunctional HPA response to TBI. Indeed, clinical evidence has shown that patients exposed to pre-injury stress or disease have significantly lower cortisol levels following TBI compared to patients who did not report stress (Sörbo et al., 2020). This could be indicative of a stress-induced reduction in HPA axis functionality, but future studies will need to investigate the precise mechanisms through which pre-injury stress affects TBI outcome.

Interestingly, recent experimental evidence suggests that prior exposure to stress before TBI can also be neuroprotective. When adolescent rodents exposed to early-life CUS are given several weeks to recover from stress before receiving TBI in adulthood, they exhibit decreased behavioral deficits compared to rodents receiving TBI with no stress (de la Tremblaye et al., 2021). In general, ELS seems to exacerbate the damage and cognitive consequences sustained by TBI but may provide some protection if followed by adequate recovery time (de la Tremblaye et al., 2021; Lajud et al., 2021; Sanchez et al., 2021). The relationship between TBI and stress is complex, and it is important to point out that chronic stress during development could have a unique impact on the response to TBI compared to pre-injury stress during adulthood. More work is needed to elucidate the mechanisms through which pre-injury stress may be either detrimental or beneficial to long term outcomes.

### 4.3 Post-injury stress impairs functional recovery

After injury, TBI survivors are highly susceptible to multiple forms of secondary stress, such as chronic pain and insomnia. Importantly, many of these post-injury stressors can also be exacerbated by recovery-related environmental stress, such as sleep loss, isolation, and medical anxiety (Jain et al., 2014; Agtarap et al., 2021). When the HPA axis has recently been activated by TBI, subsequent activation by other stressors can result in an impaired stress response (reviewed by Komoltsev and Gulyaeva, 2022). This impaired response during the vulnerable post-injury period can result in loss of essential immediate anti-inflammatory actions of GC, exacerbating the chronic inflammatory state (Komoltsev and Gulyaeva, 2022). Additionally, survivors of TBI have an increased likelihood of developing post-traumatic stress disorder (PTSD), which can amplify the stress and trauma experienced post-TBI (Spadoni et al., 2018). Chronic variable stress (CVS) is frequently used to model PTSD in rodents, and CVS followed by TBI causes greater cognitive deficits than either CVS or TBI alone (Fesharaki-Zadeh et al., 2020). A recent review of clinical PTSD literature estimated that 13.5% of non-military mild TBI survivors develop PTSD, and in many cases these symptoms progressively worsen over time (Van Praag et al., 2019). TBI survivors also have increased probability of insomnia, and clinical evidence suggests that post-injury insomnia is correlated with HPA dysfunction in patients with mTBI (Zhou et al., 2016; Zhou and Greenwald, 2018). Experimental studies in mice demonstrate that 3 days of post-injury sleep disruption increases TBI-induced neuroinflammation (Tapp et al., 2020). When post-TBI sleep fragmentation is extended to 30 days, mice exhibited hippocampal dysfunction and deficits in memory acquisition (Tapp et al., 2022). Notably, this was also associated with increased cortical expression of stress- and inflammation-related gene and inhibition of upstream regulation by *Nr3c1*, suggesting that post-TBI sleep fragmentation stress suppresses anti-inflammatory actions of GR (Tapp et al., 2022).

Preclinical evidence from studies coupling multiple experimental TBI models with various post-injury stress paradigms similarly support that post-injury stress exacerbates both cognitive and physiological consequences of TBI. In a repetitive concussive TBI model in rats, post-injury foot shock stress resulted in significantly worsened depressive-like behavior than TBI alone (Klemenhagen et al., 2013). Several weeks of post-injury social isolation exacerbated cognitive outcomes and hippocampal apoptosis in a rat penetrating TBI model (Khodaie et al., 2015). A week of restraint stress after moderate TBI in a CCI mouse model caused increased endoplasmic reticulum (ER) stress and autophagy, resulting in increased neuronal loss (Gao et al., 2022).

These studies suggest that post-injury stress exacerbates TBI-induced dysfunction, inhibiting functional recovery. Together, this emphasizes the need for TBI therapeutics that take into consideration the effects of secondary stress, though much more work is needed to dissect the mechanisms through which stress affects long-term recovery after TBI.

## 5 Previous strategies for post-TBI GC and GR therapeutics

Synthetic GCs such as dexamethasone and methylprednisolone have been used for their immunosuppressive and anti-inflammatory effects for decades, and previous clinical studies have explored their potential as a therapeutic for treating TBI (key clinical studies are summarized in Table 1; Alderson and Roberts, 2005). Synthetic GCs mimic the actions of endogenous GCs and bind GR, inducing rapid effects through membrane-associated GRs or delayed effects through translocation of GR to the nucleus. Like endogenous GC signaling, these actions regulate transcription of downstream anti- and pro-inflammatory effectors.

**Table 1:** Clinical studies on glucocorticoid usage after TBI.

	**Glucocorticoid dosage**	**Post-injury time of treatment**	**Outcome**	**Conclusion**
Gobiet et al. (1976)	Dexamethasone; “normal” dose (16 mg/day) or high dose (48–96 mg/day) for up to 8 days	Started day of TBI	Significant reduction in mortality and improvements in ICP with high dose, No significant effects of lower dose	High dose of GC improves mortality
Faupel et al. (1976) and Faupel (1982)	Dexamethasone; low dose (initially 14 mg/day, then tapered down) or high dose (initially 100 mg/day, then tapered down)	Started day of TBI	Reduced mortality and improvement in symptoms of midbrain damage (decerebrate rigidity, paresis) in high dose dexamethasone group	Early administration of high doses of GC improves mortality and neurological recovery
^*^Saul et al. (1981)	Methylprednisolone; 200 g initial dose followed by 125 mg every 6 h for 7–10 days	< 6 h after injury	Some improvements in neurologic recovery, moderate increase in survival rate	No significant effect of steroid treatment
Sugita et al. (1983)	Dexamethasone; 8–10 mg twice weekly until total dose of 80–100 mg was reached	3–15 months post-TBI	Six of the 14 cases discussed showed varying degrees improved vision; some patients had also previously used other therapeutics or medications, including oral steroids	Dexamethasone can treat post-traumatic vision impairment in some cases
^*^Braakman et al. (1983)	Dexamethasone; 100 mg initially, then tapered over 10 days	< 6 h after injury	No significant difference in survival rate at 1 month or overall outcome after 6 months; increased, but not statistically significant (*p* = 0.07), incidence of pulmonary infection with treatment	No significant effect of steroid treatment
Giannotta et al. (1984)	Methylprednisolone, high dose (30–250 mg every 6 h) or low dose (1.5–25 mg every 6 h)	6 h after injury	No significant difference between low dose group and placebos. Reduced mortality in high dose group, but this was associated with negative side effects	High doses of GC can improve mortality rate after TBI but have increased risk of negative side effects
Jackson and Mysiw (1989)	1 mg dose of dexamethasone (used for DST)	2–10 months post-TBI	Majority of patients exhibited HPA dysfunction, and 34/35 exhibited non-suppression response to DST, but DST could not predict response to TCA	HPA dysfunction is prevalent after TBI
^*^Gaab et al. (1994)	Dexamethasone; 500 mg initial dose, then 200 mg 3 h later, then another 200 mg every 6 h for eight doses	< 3 h after injury	No statistically significant differences between treatment groups	No significant effect of steroid treatment
^*^Grumme et al. (1995)	Triamcinolone acetonide; 200 mg initially, then tapered over 8 days	< 4 h after injury	Improvement in survival in steroid treatment group, but not statistically significant; more significant improvements in patients with focal lesions	Steroid treatment improves recovery from focal lesions, but no significant effect on survival
Watson et al. (2004)	Specific GC varied by subject: Most (98%) received dexamethasone but dosage varied (from 20 to ≥160 mg)	0–7 days post-TBI	GCs did not reduce development of post-traumatic seizure; Patients taking GCs within 1 day of TBI were more likely to develop seizures than those taking no GC	Early steroid treatment increases risk of post-traumatic seizures
^*^CRASH trial collaborators (Edwards et al., 2005)	Methylprednisolone; 2 g for 1 h followed by 0.4 mg for 48 h	< 8 h after injury	Increased mortality in GC treated group compared to control, at both 2 weeks and 6 months	Corticosteroids should not be used for treatment of head injury

GC dosages and outcomes of previous clinical studies are listed. Major randomized control trials are indicated with an asterisk. Overall, some clinical studies suggested improvement with GC treatment after TBI, but many randomized clinical trials have reported no significant effects. Some studies have suggested that GC use worsens outcome after injury.

### 5.1 Issues with previous synthetic GC therapeutics

Early clinical reports noted the efficacy of GCs in reducing ICP and edema, resulting in significant neurological improvements in patients with brain tumors (F'rench and Galicich, 1964). Soon, GCs were more widely used to relieve ICP in patients with other neurological conditions, including severe head trauma (Pickard and Czosnyka, 1993; Cook et al., 2020). Unfortunately, the ability of GCs to affect ICP in patients suffering TBI seems to be more limited. Despite promising preclinical results, multiple clinical studies have failed to demonstrate significant effects on functional recovery with glucocorticoid treatment after TBI (Braakman et al., 1983; Hoshide et al., 2016). Early clinical trials suggested that GCs increased survival after TBI, but the results of multiple randomized control trials suggest no significant effect of the drugs (Grumme et al., 1995; Olldashi et al., 2004; Edwards et al., 2005). In fact, the largest clinical trial, known as the corticosteroid randomization after significant head injury, or CRASH trial, reported increased mortality with corticosteroid treatment compared to controls (randomized control trials are summarized in Hoshide et al., 2016 and indicated with an asterisk in Table 1; Edwards et al., 2005; Hoshide et al., 2016). The inability of many of these studies to reach statistical significance leads to speculation that the timing and dose of GC administration may be key (Hoshide et al., 2016). Additionally, this raises the question of whether the methods of GC administration may be highly case-dependent, as severity and type of TBI vary from patient to patient. This would explain why preclinical studies, with model organisms in a controlled environment and consistent injury severity between individuals, generate significant results while clinical studies consistently fail to reach significance.

More recently, GCs have been studied in preclinical models for other post-injury injury applications, such as stabilization of the BBB and alleviation of neuroinflammatory edema (Hue et al., 2015; Moll et al., 2020). A clinical case study from 2021 examined nine patients who were given steroids to manage delayed cerebral edema after mild TBI (Prasad, 2021). All patients exhibited improvement of symptoms over a 2 year period, suggesting steroids can be safely used at lower doses in some cases. However, outcomes of steroid use after TBI remain inconsistent, and some studies suggest that high doses aggravate injury and impair cognition (Chen et al., 2009, 2011). Additionally, long-term use of GCs is associated with many negative side effects, including diabetes and osteoporosis (Marzbani and Bhimaraj, 2022). Many of these side effects are due to systemic immunosuppression that occurs over time with GC use, and systemic infection has been reported in clinical studies even at lower GC doses (Grumme et al., 1995). Interestingly, early GC use after TBI is also associated with increased risk of post-traumatic epilepsy, which may be due to the association of GR with calcium channels in the hippocampus (Karst et al., 1999; Watson et al., 2004). This emphasizes the need to better understand both the non-genomic and genomic mechanisms of GR after TBI and highlights the many issues with previous GC therapeutics.

### 5.2 Limited translational potential of GR antagonism

With the mixed success and potential risks associated with corticosteroid treatment, experimental studies have explored other avenues of manipulating endogenous corticosteroid signaling. Furthermore, excessive GC concentrations have been shown to be neurotoxic, resulting in increased neuronal sensitivity to injury through overactivation of GR (Sapolsky, 1985; Mccullers et al., 2002a). As such, GR has been the subject of many recent studies, in a variety of different stress and injury contexts, with the goal of manipulating GR functionality without triggering these negative consequences. The GR antagonist mifepristone (RU486) is frequently used in rodent models of TBI and has shown some promise in improving post-injury outcomes. Pretreatment with mifepristone was shown to protect against hippocampal neuronal loss in a rat model of CCI (Mccullers et al., 2002a). Hippocampal damage has previously been implicated in development of psychiatric symptoms after TBI, and more recent work has explored the role of GR in post-injury cognitive and psychological outcomes (Meyer et al., 2012). Indeed, mifepristone pretreatment has been shown to prevent anxiety-like symptoms after mild TBI in rats (Fox et al., 2016). Mifepristone also counteracts some of the negative effects of treatment with the corticosteroid dexamethasone after TBI (Zhang et al., 2020). These studies provide evidence that neurotoxic effects of high GC levels act through GR and suggest that blocking GR signaling could improve recovery. However, the progesterone receptor-mediated effects of mifepristone on pregnancy, as well as additional adverse side effects such as liver toxicity, limit its translational potential and create a need for alternative approaches to GR manipulation (Meyer et al., 1990; reviewed by Nieman, 2022).

## 6 Mechanisms of GR action are context-dependent

As a transcriptional regulator, GR affects upregulation and downregulation of hundreds of targets with a wide range of downstream consequences. In general, anti-inflammatory actions of GR involve silencing of inflammatory genes, while pro-inflammatory GR effects involve direct binding and upregulation of inflammatory targets. Recent studies suggest that both mechanisms of action rely on direct DNA binding of GR to its targets (Escoter-Torres et al., 2020). Once inside the nucleus, the downstream effects of GR transcriptional activity are largely context-specific. The GREs to which GR binds depend on chromatin structure and DNA accessibility, which varies between cell types (John et al., 2011). Coregulator proteins are also recruited when GR binds to GREs, acting together with GR to repress or activate transcription of target genes. This repertoire of coregulators influences the downstream outcomes of GR signaling, and they vary from cell to cell, further lending to the cell-specificity of GR activity (Weikum et al., 2017; Sacta et al., 2018). GR is expressed on almost every cell throughout the body, but the level of expression and the alternative isoforms expressed vary by tissue type (Turner et al., 2006). Differences in cell type-specific repertoire of regulatory proteins and chromatin structure create distinct environments that enable context-dependent downstream effects of GR throughout the body (Zalachoras et al., 2016). We propose that a better understanding of this context-specific functionality of GR may inform development of future therapeutics. A more tailored, cell-specific approach to targeting GR may yield better results than previous methods with global GCs and GR antagonists.

### 6.1 Rodent models reveal essential functions of GR

Transgenic rodent models are powerful tools for studying the functions of conserved genes. However, *Nr3c1* is an essential gene in mammals, so there are no viable GR-null rodent strains (Cole et al., 1995). As an alternative approach, antisense RNA was used to create transgenic mice with decreased expression of GR, resulting in significant HPA axis dysfunction and cognitive impairment (Pepin et al., 1992; Montkowski et al., 1995; Barden et al., 1997). A major caveat of this model is that it only creates a partial loss of GR function, limiting its applications for molecular studies of GR signaling. Additionally, the global and constitutive knockdown of GR makes it difficult to distinguish developmental effects of GR loss from acute GR functions in adult mice. The use of this antisense RNA model has declined, and it is rarely used today.

More recently, tissue-specific functions of GR have been investigated using Cre-loxp recombination. In this system, Cre recombinase is expressed under a tissue-specific promoter, and a gene of interest is flanked by loxP sites. In cells expressing Cre recombinase, the loxP flanked (“floxed”) gene is excised, generating a tissue-specific deletion. This Cre-loxp system has been used to delete GR in the brain and peripheral cells, revealing novel insight into the tissue-specific role of GR (Tronche et al., 1999; Arnett et al., 2011; Liu et al., 2021). We will focus on the function of GR in the brain.

### 6.2 GR mechanisms are brain-region dependent

GR is abundantly expressed throughout the brain, crucial for maintaining homeostasis and facilitating recovery in response to stress (De Kloet et al., 2005; Joëls, 2018). Previous studies also suggest that GR in the brain plays a key role in learning and memory (Oitzl, 1997; Oitzl et al., 2001; Steckler et al., 2001). The role of GR in memory-related tasks has especially been demonstrated in the context of experimental studies associated with stress. For example, the Morris water maze task is commonly used to assess spatial memory (Vorhees and Williams, 2006). The water maze task has been shown to evoke a stress response in mice, and this significantly affects learning and memory when glucocorticoid signaling is impaired (Aguilar-Valles et al., 2005; Lengel et al., 2022). Cre-loxp deletion of GR in mouse neurons and glial cells leads to dysfunctional HPA axis feedback regulation and significantly elevated HPA activity, similar to symptoms of Cushing syndrome in humans (Tronche et al., 1999). This highlights that nervous system expression of GR is required for normal stress response functionality.

GR is highly enriched in structures of the limbic system, which regulates multiple processes to maintain homeostasis such as emotion, learning, memory, and motivation (Torrico and Abdijadid, 2023). This includes the hippocampus, amygdala, and hypothalamus (Erdmann et al., 2008). One of the first localized GR knockout mouse models was a forebrain-specific GR knockout, which disrupted GR in multiple essential limbic system structures (Boyle et al., 2004, 2006). Importantly, this model resulted in delayed loss of GR completing around 4–6th months, allowing for exclusion of developmental effects of GR deletion. This deletion resulted in disruption of circadian HPA activity, as well as an increase in depressive-like behavior (Boyle et al., 2004). An amygdala-specific deletion of GR was later generated by injecting lentiviral vector expressing Cre recombinase directly into the central amygdala in GR loxP mice (Kolber et al., 2008). These mice exhibited significant deficits in contextual and auditory-cued fear conditioning, suggesting a role for amygdala GR in learning and memory (Kolber et al., 2008; Arnett et al., 2011).

Within the hippocampus, GR is highly expressed in the CA1, CA2, and dentate gyrus, with lower expression in the CA3 region (Fuxe et al., 1985; Sarabdjitsingh et al., 2009). One of the major functions of GR in the hippocampus involves its association with membrane receptors, such as voltage-dependent calcium channels and hyperpolarization-activated cyclic nucleotide-gated channels (Kerr et al., 1992; Kim et al., 2022). Activated GR modulates currents through these membrane-channels, a key component of regulating neuronal excitability and maintaining homeostasis under stress (Kerr et al., 1992; Zü and Reiser, 2011; Lyman et al., 2021; Kim et al., 2022). Region specificity is seen in GR activity even within the hippocampus. Previous studies on GR-dependent calcium channel activity show differential effects of corticosterone on calcium currents in the CA1 and dentate gyrus (Chameau et al., 2007; Van Gemert et al., 2009). Interestingly, transcriptional analysis showed no difference in GR-associated transcriptional control of calcium channels (Van Gemert et al., 2009). This highlights the complex region specificity of GR signaling, and demonstrates the importance of considering both genomic and non-genomic effects of GR.

### 6.3 Brain region-dependent effects of TBI

The pathophysiology of TBI is highly varied. The location and severity of the initial impact, as well as the degree of diffuse vs. focal injury, can result in differing levels of damage to multiple different brain regions (McGinn and Povlishock, 2016). Clinical and experimental evidence shows that the hippocampus and other limbic system tissues are particularly vulnerable to structural and inflammatory damage following TBI, though the precise region-specific consequences vary with injury type and severity (Christensen et al., 2020; Drieu et al., 2022). The effects of TBI on calcium channel-related activity of GR in the hippocampus in particular are worth investigating, as this could relate to mechanisms of post-TBI excitotoxicity (Chameau et al., 2007). Still, it would be interesting to study how the role of GR differs in other brain regions affected by TBI, such as the cortex. Ostensibly, GR-mediated effects of TBI are heavily dependent on the brain regions affected by injury, though future studies will need to more closely explore these region-dependent effects.

### 6.4 TBI-dependent GR mechanisms require further investigation

In addition to region- and cell-specific GR functions, the GR-mediated response to stress is also heavily influenced by the type and severity of the stressor. This is important because the primary and secondary phases of injury, as well as the extended period of chronic recovery, exert very different physiological and stress-related effects. To date, few studies have directly analyzed GR/*Nr3c1* response to TBI. Many of the studies that report effects of TBI on stress signaling through GR and MR make use of receptor antagonists like mifepristone to assess receptor function. The results of a 2002 preclinical CCI study in rats indicated inhibition of GR mRNA 24 h after injury in the hippocampus and dentate gyrus (Mccullers et al., 2002b). Pretreatment with mifepristone prior to injury did not result in increased GR mRNA, suggesting that TBI impaired negative feedback regulation of GR (Mccullers et al., 2002b). A 2022 study involving FPI in mice reported that sleep fragmentation for 30 days after injury led to inhibition of Nr3c1 regulation (Tapp et al., 2022). An experimental study from last year reported a decrease in hippocampal *Nr3c1*, quantified using qPCR, in male rats more than 80 days after FPI (Ju et al., 2023). These recent studies point to chronic inhibition of GR/*Nr3c1* after TBI. The earlier results from Mccullers et al. suggest impaired negative feedback after TBI, which resulted in decreased GR mRNA 24 h after injury. Still, more work is needed to dissect the mechanisms of GR that contribute to altered stress signaling at both acute and chronic time points. Future studies that distinguish between the different isoforms of GR, as well as the genomic and non-genomic effects, will help shed light on the role of GR in HPA dysfunction after TBI. GR has been studied more extensively in the contexts of other stressors, and there are distinct responses to acute and chronic stress (Li et al., 2019). The response of GR in these other stress contexts could inform future studies on the effects of primary and secondary brain injury on GR signaling.

### 6.5 GR activation in the brain after acute stress

Preclinical studies have shown GR activation in response to acute psychological stress, where acute stress is defined as a rapid intense exposure to stress. Restraint stress has long been used as a model of psychological stress, and studies suggest distinct GR-mediated effects that vary by timing and duration of restraint. Within the hippocampus, a single 20-min exposure to acute restraint in mice induces a small but significant transient decrease in total mRNA expression of *Nr3c1*, the gene encoding GR (Viudez-Martínez et al., 2018). Another study showed increased phosphorylation of GR following 1 h of acute restraint, which may affect downstream activity of GR (Papadopoulou et al., 2015). A similar acute restraint paradigm in rats showed a decrease in cytosolic GR but increase in nuclear GR in hippocampus following restraint stress (Green et al., 2016). GR translocates to the nucleus upon activation, and these studies together suggest a decrease in total expression but increase in activated GR following acute restraint. Forced swim is another psychological stress paradigm in rodents, and a single 15-min forced swim trial results in significant upregulation of several GR target genes in the hippocampus, indicative of increased GR activity (Mifsud and Reul, 2016). Together, these studies indicate increased activation of GR following acute stress.

The inflammatory stimulus lipopolysaccharide (LPS) has been widely used to induce acute immune challenge and neuroinflammatory response. Microglia are highly sensitive to stress-induced changes in the brain environment, and studies using microglial GR-depleted mice suggest that GR in microglia is neuroprotective following LPS stimulus, preventing LPS-induced neurodegeneration and repressing expression of Kv1.3 calcium channels (Carrillo-De Sauvage et al., 2013).

### 6.6 Alterations in GR activity in chronic stress

Chronic exposure to stress, where the stressor endures for an extended period or occurs repeatedly over multiple days, induces changes in HPA axis functionality, altering the role of GR as stress persists. Preclinical and clinical evidence indicates that prolonged stress or injury can cause HPA axis dysfunction, resulting in functional differences in GR signaling. Endocrine disorders such as Cushing's disease and diabetes mellitus can directly affect GR expression and function (Mu et al., 1998; Panagiotou et al., 2021). Other conditions like cardiovascular disease and obesity are associated with chronic disruption of HPA feedback regulation and disrupted GR sensitivity (Ljung et al., 2002). Preclinical models of chronic stress show distinct differences between effects of acute and chronic stress on HPA function and GR signaling. Chronic restraint stress, for example, has been shown to cause downregulation (Chiba et al., 2012; Viudez-Martínez et al., 2018). Chronic unpredictable stress, in which rodents are exposed to various factors such as forced swimming, physical restraint, and food or water deprivation, induces progressive downregulation of hippocampal *Nr3c1* (Li et al., 2020). This effect is more pronounced over time, with Nr3c1 expression decreasing over several weeks as mice are continually exposed to stress (Li et al., 2020).

When mice receive an additional LPS challenge following chronic stress, inhibition of GR with the receptor antagonist RU486 significantly prevented LPS-induced neuronal damage. This suggests that in the context of chronic stress, GR is a key mediator of inflammation and neurodegeneration. This is in stark contrast to the neuroprotective role of GR that has been observed in response to acute LPS exposure alone (Espinosa-Oliva et al., 2011; Carrillo-De Sauvage et al., 2013). Taken together, this highlights that even within the brain, the downstream activity of GR signaling is highly context dependent.

## 7 New approaches for GC therapeutics in TBI

With the controversial results of previous studies, and the high potential for adverse side effects, GC use in TBI patients during recovery has understandably dwindled. However, the promising anti-inflammatory effects of glucocorticoids have continued to encourage studies aimed at exploiting the benefits of GC treatment while circumventing the negative side effects. Recent preclinical work has investigated the use of dexamethasone-containing hydrogels for local controlled delivery of GC at the site of injury (Jeong et al., 2021; Macks et al., 2022). Hydrogel treatment significantly reduced inflammation and improved functional recovery in a rat CCI TBI model, and local delivery avoids risk of systemic side effects (Macks et al., 2022). Another strategy that has emerged is the use of macromolecular GC prodrugs. GC prodrugs have been applied in other disease contexts, such as inflammatory arthritis, and have been shown to greatly improve the efficacy of GC, limiting systemic exposure and reducing side effects commonly associated with long-term GC use (Jia et al., 2020; Zhao et al., 2021). A recent experimental study using a dexamethasone prodrug (P-Dex) demonstrated significant improvement of neuroinflammation and neurodegeneration after 14 days of systemic delivery, correlating with increased functional outcomes, in a mouse CCI model (Wei et al., 2022). It is worth noting that under normal circumstances, macromolecular therapeutics like P-Dex are unable to enter the blood brain barrier, restricting their use in diseases of the CNS (Zhao et al., 2016). However, in this mouse TBI model, the compromised brain vasculature enabled P-Dex to sufficiently enter the brain and target the injured tissue (Wei et al., 2022). The authors speculate that similar BBB disruption would allow for macromolecular drug delivery in other models, including human TBI patients. However, this is a very new drug delivery strategy, and much work remains to be done to establish its efficacy in TBI (Zhao et al., 2016; Cash and Theus, 2020).

### 7.1 Genetic manipulation of GR could reveal context-specific responses to TBI

As GR is ubiquitously expressed and has highly context- and tissue-specific roles throughout the body, global manipulation with a receptor antagonist such as mifepristone has variable effects in different cell types throughout the body. This could complicate interpreting results from studies like these and make it difficult to generate clear results on cognitive and behavioral outcomes. Genetic approaches for tissue- or cell-specific GR manipulation will likely a better option going forward.

Using GR-expressing lentivirus, a recent preclinical study found that GR overexpression in the dorsal hippocampus improved cognitive outcomes after pediatric TBI (in a CCI model) in rats (Lengel et al., 2022). Additionally, it was found that TBI caused decreased mRNA expression of the GR target *Sgk1* compared to controls (Lengel et al., 2022). This suggests that TBI-induced impairments in hippocampal GR functionality may underlie post-injury cognitive impairment. Interestingly, this seems to contradict what has previously been reported with mifepristone treatment, where blocking GR was beneficial to TBI outcome. It could be that pediatric and adult TBI have differential effects on GR signaling. With the availability of Cre-loxp rodent models, and advancements in gene therapy techniques such as lentiviruses, it is worth looking at tissue- and developmental stage-specific effects of GR on TBI outcomes.

### 7.2 The role of microglial GR in TBI

As the resident immune cells of the nervous system, microglia play a fundamental role in sensing and responding to changes in the brain environment to maintain homeostasis. Microglia undergo morphological and functional changes when exposed to stress and are an essential component in the brain's response to injury. However, prolonged activation of microglia perpetuates neuroinflammation and contributes to neurodegeneration. Clinical and preclinical evidence suggest chronic post-injury changes in microglia, including the development of primed microglia, after TBI (Fenn et al., 2014; Krukowski et al., 2021; Witcher et al., 2021; reviewed by Wangler and Godbout, 2023). Primed microglia are hypersensitive to secondary stress or immune challenge, mounting an exaggerated inflammatory response associated with cognitive deficits and psychiatric dysfunction (Muccigrosso et al., 2016). Depletion of injury-associated microglia after TBI reduces neurodegeneration and neurological deficits, highlighting the important role of microglia in mediating post-injury outcomes (Witcher et al., 2018; Henry et al., 2020; Bray et al., 2022).

GR is highly expressed on microglia and is a key link between the stress response in the brain and the microglia-mediated inflammatory response (Sierra et al., 2008). Given the instrumental role of microglia in chronic neuroinflammation, microglia-related GR signaling has been a key subject of study in recent preclinical models of stress and injury.

Healthy aging is associated with heightened neuroinflammation and exaggerated response to stress or immune challenge, and experimental evidence has shown that this is mediated by primed microglia (reviewed by Norden and Godbout, 2013). When rats are administered intracranial mifepristone, microglial pro-inflammatory responses are significantly diminished. Furthermore, the immune response to *E. coli* infection was reduced, and infection-associated memory deficits were rescued (Barrientos et al., 2015). These findings suggest that GR facilitates microglial priming in the context of aging. In another study, GR antagonism with mifepristone prevented microglia-mediated neuronal remodeling and behavioral despair following chronic unpredictable stress (CUS) in mice (Horchar and Wohleb, 2019). This suggests that GR facilitates phagocytic behavior and neuronal interactions of microglia after CUS and supports the notion that GR may mediate microglia activation.

Genetic deletion of GR in myeloid cells, including microglia, improves recovery in a mouse model of spinal cord injury (Madalena et al., 2022). This was correlated with impaired microglia and macrophage infiltration of the injury site, as well as reduced activation of these cells (Madalena et al., 2022). It is worth pointing out that these results are seemingly contradictory to the improvements in cognition that were reported with hippocampal overexpression of GR in rat TBI (Lengel et al., 2022). This emphasizes the highly context dependent role of GR. The type of injury or stress, as well as the cell and tissue type, all play a role in determining the downstream outcomes of GR signaling. Furthermore, Madalena and colleagues pointed out that depletion of GR from myeloid cells could result in compensatory mechanisms in GR signaling from other cell types (Madalena et al., 2022). Future studies are needed to determine these mechanisms, and it would be interesting to see if the role of GR is similar in the context of other forms of CNS trauma.

A 2022 study uncovered a role for GR in modulating outcomes in a model of rodent food restriction (FR) prior to TBI (Perović et al., 2022). Pre-injury FR was previously shown to increase levels of circulating GC and suppress TBI-induced microglial activation (Lončarević-Vasiljković et al., 2009; Loncarevic-Vasiljkovic et al., 2012). In this newer study, it was found that FR enhances nuclear localization of GR and induces upregulation of downstream GR targets (Perović et al., 2022). This suggests that the neuroprotective effects of pre-TBI FR may act through increased GR signaling. Further analysis is needed to determine the cell-specific role of GR in this model, and to confirm whether this increased GR affects microglia. Nevertheless, these recent studies together suggest GR may be an important link between the stress response and microglia-mediated pathophysiology in multiple contexts.

## 8 Conclusions and future directions

We have established that there is a complex, intertwined relationship between stress and TBI, both pre- and post- injury. We have also discussed that TBI induces robust neuroendocrine dysfunction, resulting in additional alterations of the stress response and further exacerbating consequences of injury. This emphasizes the need to address stress functionality after TBI, especially during chronic recovery.

Recent evidence points to GR in microglia-mediated outcomes of stress and injury, playing a role in microglia priming and activation. Primed microglia have also been shown to underlie much of the chronic neuroinflammation that persists after TBI, worsening cognitive and functional outcomes. Importantly, TBI survivors are highly vulnerable to additional stress as they recover after injury. TBI also induces robust neuroendocrine dysfunction, meaning that TBI survivors experience impaired response to stress after injury. This can further exacerbate TBI-induced neuroinflammation and neurological deficits and complicate recovery. This period of chronic recovery makes up the vast majority of post-injury time for most survivors. It is critical that therapeutics be developed to understand and address these chronic stress and inflammation related mechanisms.

GCs have been extensively explored as an anti-inflammatory TBI therapeutic. However, prolonged GC treatment is associated with high risk of adverse side effects. Furthermore, with some studies showing that high doses of GC are neurotoxic and aggravate injury, it seems the risks are no longer worth the potential benefits of GC treatment. Recent developments in biomedical engineering and drug delivery systems are currently being applied to administer local controlled delivery of GC, but these are very new developments and there is much work to be done to determine whether these will be effective approaches.

Manipulation of GR is a promising avenue of research that can yield the anti-inflammatory benefits of GC therapeutics without the neurotoxic and systemic effects of steroid treatment. However, few studies so far have examined this in the context of TBI. Results from experimental studies in other injury and stress contexts, including CUS and spinal cord injury, implicate GR in microglia-mediated pathophysiology and suggest that GR plays a role in microglia activation. In this review, we discussed how highly context- and tissue-specific mechanisms of GR action are, and thus it is difficult to generalize the role of GR after injury. It will be important in future studies to explore cell-specific roles of GR in the context of TBI. Future studies should consider how GR in microglia and neurons influences long-term recovery after TBI, especially when stress is involved. Additionally, it may be worth delineating the rapid vs. delayed mechanisms of nervous system GR in response to neurotrauma. Considering the important role of membrane-associated GR in the hippocampus, non-genomic mechanisms may play a big role in pathology in the brain. It will be interesting to investigate both the non-genomic and genomic downstream consequences of GR signaling in the brain, with special attention to microglia-mediated consequences of TBI. With the availability of transgenic rodent models and novel gene editing techniques, we have valuable tools available to investigate the context-specific mechanisms of GR in modulating outcomes after TBI in the near future.

## Author contributions

MT: Conceptualization, Writing—original draft, Writing—review & editing. OK-C: Conceptualization, Writing—review & editing.
